# A transgenic in vitro cell model for the analysis of proinflammatory effects of naturally occurring genetic variants of caspase-1

**DOI:** 10.1186/1546-0096-13-S1-P18

**Published:** 2015-09-28

**Authors:** F Schulze, E Hengst, S Winkler, A Rösen-Wolff

**Affiliations:** 1 University Hospital Carl Gustav Carus, Department of Pediatrics, Dresden, Germany

## Introduction

Caspase-1 (Interleukin-1 Converting Enzyme, ICE) is a proinflammatory enzyme mediating cleavage and secretion of the proinflammatory cytokines IL-1β and IL-18. Caspase-1 plays pivotal roles in the innate immune system and in inflammatory diseases like periodic fever syndromes, arthritis, or type-II-diabetes. In previous studies published by our group, genetic variants of procaspase-1 had been detected in patients suffering from autoinflammatory symptoms. Analyses of the procaspase-1 variants in HEK293T cells revealed reduced enzymatic activity of caspase-1 but enhanced ability to activate NF-κB signaling. The latter was mediated by enhanced CARD/CARD interactions of procaspase-1 with RIP2.

## Objectives

The primary objective of this study was to analyze the effects of procaspase-1 variants in a monocyte/macrophage cell model. Furthermore, protein expression and enzymatic activity of procaspase-1 with or without a C-terminal FLAG-tag was analyzed.

## Materials and methods

Genetically engineered THP-1 monocytes were used for the *in vitro* study. First, THP-1 cells were transduced with lentiviral vectors expressing shRNA against procaspase-1 mRNA. Subsequently, procaspase-1 wildtype (wt) or variants with or without a C-terminal FLAG-tag were reconstituted using a second lentiviral transduction. THP-1 cells were differentiated into macrophages and stimulated with different inflammasome activators. Activation and release of caspase-1 and IL-1β was assessed using immunoblotting (caspase-1) or immunoblotting and cytometric bead arrays (IL-1β). The activation of NF-κB was estimated by measuring the NF-κB regulated cytokines IL-6 and IL-8. Cell death following inflammasome activation was analyzed by measuring LDH in the cell culture supernatant.

## Results

The protein expression level of reconstituted FLAG-tagged procaspase-1 variants was significantly reduced compared to expression of endogenous procaspase-1 or reconstituted procaspase-1 variants without FLAG-tag. In line with this data, the release of IL-1β after inflammasome stimulation was reduced in cells expressing FLAG-tagged procaspase-1 compared to cells expressing procaspase-1 without FLAG-tag. Interestingly, the mRNA expression of the FLAG-tagged procaspase-1 variants was not reduced compared to reconstituted procaspase-1 without FLAG-tag. Furthermore, cells expressing the procaspase-1 variants released reduced amounts of IL-1β and showed a reduced frequency of cell death following inflammasome stimulation. No differences in IL-6 or IL-8 secretion were detected when cells expressing wt or enzymatically inactive procaspase-1 were compared.

## Conclusion

This study shows that even short protein-tags can influence protein expression significantly. Therefore, phenotypic rescue in knockdown studies can be complicated when using tagged proteins. Using the genetically engineered THP-1 cells we were able to show a reduced IL-1β release and reduced frequency of pyroptosis following inflammasome stimulation without detecting any differential regulation of the proinflammatory cytokines IL-6 or IL-8.

This study was supported by the German Research Foundation (DFG, KFO 249) and by a MeDDrive project (University of Technology, Medical Faculty) to SW.

